# Personalized First-Line Treatment of Metastatic Pancreatic Neuroendocrine Carcinoma Facilitated by Liquid Biopsy and Computational Decision Support

**DOI:** 10.3390/diagnostics11101850

**Published:** 2021-10-07

**Authors:** Judita Szkukalek, Róbert Dóczi, Anna Dirner, Ákos Boldizsár, Ágnes Varga, Júlia Déri, Dóra Lakatos, Dóra Tihanyi, Barbara Vodicska, Richárd Schwáb, Gábor Pajkos, Edit Várkondi, István Vályi-Nagy, Dorottya Valtinyi, Zsuzsanna Nagy, István Peták

**Affiliations:** 1 Department of Clinical Oncology, St. Imre Hospital, 1115 Budapest, Hungary; tjudit84@yahoo.com (J.S.); valtinyid@gmail.com (D.V.); onkologia@szentimrekorhaz.hu (Z.N.); 2 Oncompass Medicine Hungary Ltd., 1024 Budapest, Hungary; robert.doczi@oncompassmedicine.com (R.D.); anna.dirner@oncompassmedicine.com (A.D.); akos.boldizsar@oncompassmedicine.com (Á.B.); agnes.varga@oncompassmedicine.com (Á.V.); julia.deri@oncompassmedicine.com (J.D.); dora.lakatos@oncompassmedicine.com (D.L.); dora.tihanyi@oncompassmedicine.com (D.T.); barbara.vodicska@oncompassmedicine.com (B.V.); richard.schwab@oncompassmedicine.com (R.S.); gabor.pajkos@oncompassmedicine.com (G.P.); edit.varkondi@oncompassmedicine.com (E.V.); 3 Centrum Hospital of Southern Pest, National Hematology and Infectology Institute, 1097 Budapest, Hungary; drvnistvan@gmail.com; 4 Department of Pharmacology and Pharmacotherapy, Semmelweis University, 1089 Budapest, Hungary; 5 Department of Pharmaceutical Sciences, University of Illinois at Chicago, Chicago, IL 60612, USA

**Keywords:** pancreatic neuroendocrine tumor, molecular diagnostics, liquid biopsy, cfDNA, large panel sequencing, clinical decision support system, everolimus, personalized oncology

## Abstract

Background: We present the case of a 50-year-old female whose metastatic pancreatic neuroendocrine tumor (pNET) diagnosis was delayed by the COVID-19 pandemic. The patient was in critical condition at the time of diagnosis due to the extensive tumor burden and failing liver functions. The clinical dilemma was to choose between two registered first-line molecularly-targeted agents (MTAs), sunitinib or everolimus, or to use chemotherapy to quickly reduce tumor burden. Methods: Cell-free DNA (cfDNA) from liquid biopsy was analyzed by next generation sequencing (NGS) using a comprehensive 591-gene panel. Next, a computational method, digital drug-assignment (DDA) was deployed for rapid clinical decision support. Results: NGS analysis identified 38 genetic alterations. DDA identified 6 potential drivers, 24 targets, and 79 MTAs. Everolimus was chosen for first-line therapy based on supporting molecular evidence and the highest DDA ranking among therapies registered in this tumor type. The patient’s general condition and liver functions rapidly improved, and CT control revealed partial response in the lymph nodes and stable disease elsewhere. Conclusion: Deployment of precision oncology using liquid biopsy, comprehensive molecular profiling, and DDA make personalized first-line therapy of advanced pNET feasible in clinical settings.

## 1. Introduction

The right choice for first-line cancer treatment is crucial, especially for patients diagnosed at an advanced stage and with poor performance status. Precision oncology, the personalized, molecularly targeted treatment of every cancer patient based on the individual genetic alterations of their tumor, is a highly anticipated approach. However, it is still mostly confined to the use of molecularly targeted therapies (MTAs) based on single companion diagnostic tests or to off-label therapies after the exhaustion of standard therapies.

Neuroendocrine tumors (NETs) are a rare and heterogeneous group of tumors with highly diverse morphologies and behaviors. Pancreatic neuroendocrine tumors (pNETs) comprise a biologically distinct subset of NETs, which behave and respond to systemic treatments differently to NETs arising elsewhere in the gastrointestinal tract [1]. 

Multiple treatment options with MTAs have recently become available for first-line therapy in many indications, including pNET. The multi-tyrosine kinase (VEGFR, PDGFR) inhibitor sunitinib is approved for pancreatic neuroendocrine carcinoma. In a phase 3 trial, patients with advanced, well-differentiated pNETs received sunitinib treatment. The median progression-free survival (PFS) was 11.4 months in the sunitinib arm and 5.5 months in the placebo arm [2]. Somatostatin analogs are commonly used to treat symptoms associated with hormone hypersecretion in neuroendocrine tumors, and their antitumor effects were demonstrated in the CLARINET trial involving 204 patients with metastatic enteropancreatic tumors of grade 1 or 2 (a tumor proliferation index [on staining for the Ki-67 antigen] of <10%) and documented disease-progression status [3]. Treatment with lanreotide was associated with significantly prolonged PFS (at 24 months: 65.1% with lanreotide (95% CI, 54.0–75.1 months) and 33.0% with placebo (95% CI, 23.0–43.3 months)). However, the study did not prove a significant difference in overall survival (OS) between the two groups. Lutathera (lutetium Lu177 oxodotreotide), a radioactive targeted compound, is approved for advanced somatostatin receptor-positive gastroenteropancreatic neuroendocrine tumors (GEP-NETs). The approval was based on the phase 3 NETTER-1 trial, where lutathera reached 30+ months of median PFS compared to 8.4 months in the octreotide arm in patients with advanced, progressive, somatostatin-receptor–positive midgut neuroendocrine tumors [4]. The mTOR inhibitor everolimus is approved to treat advanced neuroendocrine tumors (NETs) originating in the lungs or gut when the cancer cells are well-differentiated, and the tumor is metastatic or cannot be removed by surgery. The RADIANT-3 study was conducted in patients with advanced pancreatic neuroendocrine tumors [5]. The median PFS was 11.0 months with everolimus as compared with 4.6 months with placebo (hazard ratio for disease progression or death from any cause with everolimus, 0.35; 95% confidence interval [CI], 0.27 to 0.45; *p* < 0.001), representing a 65% reduction in the estimated risk of progression or death. Everolimus treatment was associated with a low rate of severe adverse events.

Moreover, several multi-receptor tyrosine kinase inhibitors, such as nintedanib [6], lenvatinib [7], and surufatinib [8], demonstrated promising efficacy in NETs. On the other hand, many common pNET mutations are not considered effectively targetable (*DAXX*, *ATRX*, *MEN1*). Progress in targeted therapies also highlights the importance of informed treatment decision-making based on the individual tumor molecular profile.

The genetic and epigenetic alterations characteristic to pancreatic neuroendocrine tumors have been recently reviewed by Tirosh and Kebebew [9]. The most important driver alterations in pNET are mutations of *DAXX*, *ATRX*, *MEN1*, *VHL*, and mTOR pathway members, along with genes involved in DNA damage repair and chromatin modification. *DAXX* (death-domain associated protein) gene encodes a multifunctional protein that is involved in transcription coregulation, chromatin remodeling, and regulation of apoptosis. *ATRX* is a member of the SWI/SNF family of chromatin remodeling proteins. It is involved in the process of transcriptional regulation, DNA repair, and telomere length regulation. Loss of ATRX function leads to genetic instability and promotes tumorigenesis [10]. Loss-of-function mutations in the tumor suppressor *MEN1* are associated with MEN1 syndrome, a disease that causes tumors in the pituitary, parathyroid, lung, and enteropancreatic endocrine tissues [11]. The *VHL* tumor suppressor gene plays a role in the oxygen-sensing pathway. Its inactivation leads to von Hippel–Lindau disease, a hereditary cancer syndrome [12]. The PI3K–AKT–mTOR pathway is a central regulatory mechanism controlling key cellular processes, such as metabolism, motility, growth, and proliferation [13]. It is one of the most frequently dysregulated pathways in cancers, supporting cancer cells’ survival, expansion, and dissemination [14]. The most prominently mutated pathway members are *PIK3CA* (Phosphatidylinositol 4,5-bisphosphate 3-kinase catalytic subunit alpha), *PIK3R1* (Phosphatidylinositol 3-kinase regulatory subunit alpha), *PTEN* (Phosphatidylinositol 3,4,5-trisphosphate 3-phosphatase and dual-specificity protein phosphatase PTEN), *AKT1* (RAC-alpha serine/threonine-protein kinase), *TSC1/2* (Tuberous sclerosis 1/2), *STK11* (Serine/threonine-protein kinase STK11), and *mTOR* (Serine/threonine-protein kinase mTOR). 

Cell-free DNA (cfDNA) genotyping has the potential to become a powerful technology of cancer genomics in monitoring advanced cancer undergoing treatment and replacing certain tumor tissue biopsies [15,16]. This may be of special interest in pancreatic cancer, where adequate biopsy is still challenging due to poor anatomic location, often resulting in limited samples; thus, a noninvasive biomarker test is still required [16,17,18]. In cancers of the pancreas, cfDNA testing is considered to be still in the experimental stage in ductal adenocarcinomas [18,19]. In neuroendocrine tumors, cfDNA is also emerging as a biomarker, although it has not been incorporated into routine clinical practice [20]. The presence of tumor-specific alterations in cfDNA and their evolution during disease progression could be detected in metastatic pNET patients, using droplet digital PCR and shallow whole-genome sequencing to detect copy number variations (CNVs) [21]. A recent large-scale study involved 320 patients with neuroendocrine neoplasms (NEN), including 165 pancreatic NET cases. NGS analysis of ctDNA from liquid biopsies defined genomic alterations in 280 samples, most frequently *TP53*, *KRAS*, *EGFR*, *PIK3CA*, *BRAF*, *MYC*, and *CCNE1* mutations [22]. 

Besides major advances in evidence-based cancer therapy, early diagnosis has been a key driving force behind reducing cancer mortality. The unprecedented worldwide occurrence of the coronavirus SARS-CoV-2 (COVID-19) pandemic has had a profound effect on the entire oncology community, especially during the spring of 2020, including the direct impact of infections and also indirect consequences through the increased burden on healthcare capacities. This led to requests to professionals in charge of chronic diseases, including cancer, to postpone diagnosis and treatment [23]. 

Digital drug-assignment (DDA) is a computational method for precision oncology [24]. DDA helps rapid clinical decisions by assigning a DDA score, the “aggregated evidence level” (AEL) to each genetic alteration and the associated targeted drugs, based on a network of evidence-based associations between all genetic alterations present in the tumor and the associated druggable targets, and targeted therapies.

Here we present the case of a patient whose diagnosis with advanced pancreas neuroendocrine carcinoma was delayed by months due to COVID-19, yet promptly applied targeted therapy based on NGS analysis of cfDNA achieved tumor responses.

## 2. Case Report

**Figure 1:**
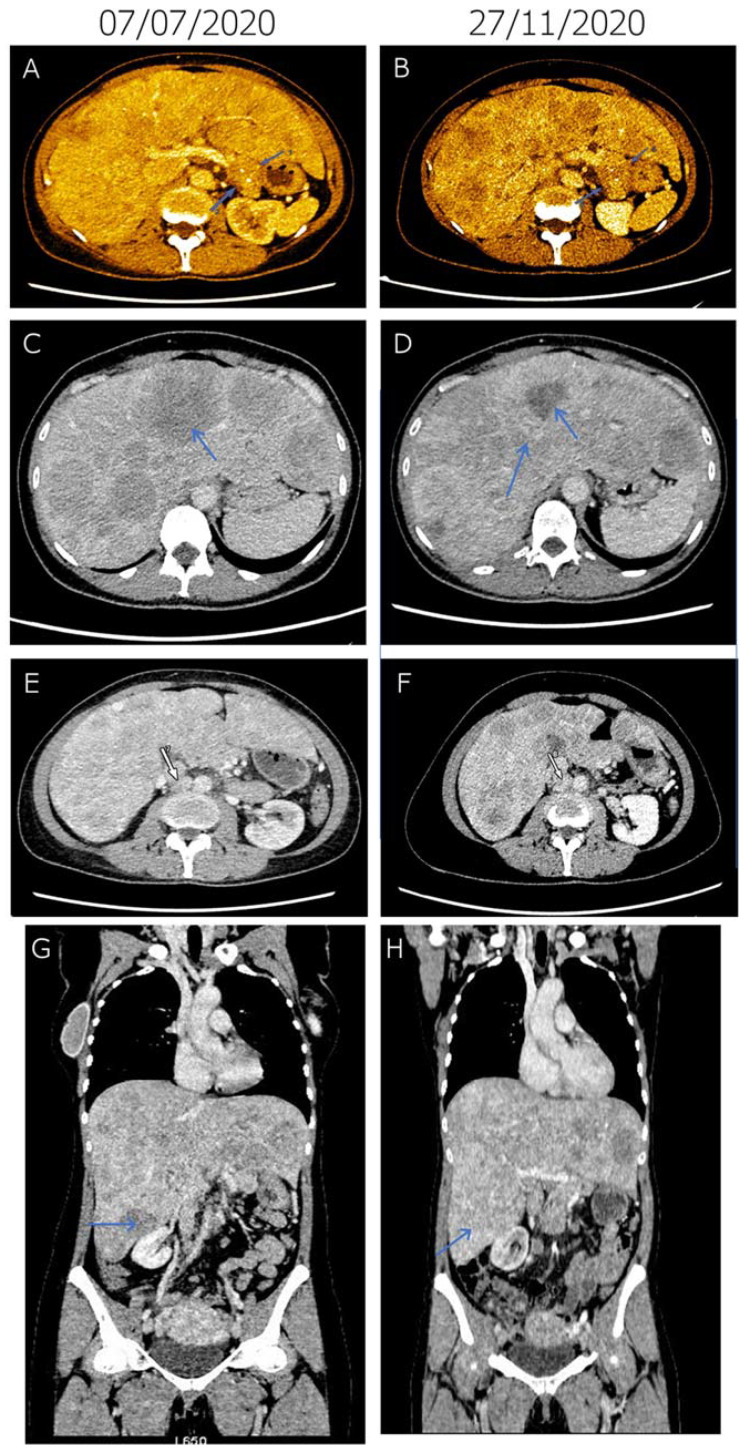
Tumor response achieved by first-line molecularly targeted everolimus therapy in advanced pancreatic neuroendocrine carcinoma. (**A**–**H**) Representative contrast CT slices at two time points: 7 July 2020 (at diagnosis, left) and 27 Novermber 2020 (right). At diagnosis (7 July 2020), a 22 × 27 mm, inhomogeneous, oval soft-tissue mass could be observed behind the pancreas tail (arrows), which could not be distinguished from the outline of the pancreas with certainty (**A**). Two tiny spots of calcification were also present. On 27 Novermber, the mass was detected with unchanged status and of the same size (arrows), indicating stable disease (**B**). At diagnosis (7 July 2020), the liver was significantly enlarged. Major parts of the liver area were filled with occasionally conflating hypodense regions of blurred outlines (arrow), indicating multiple metastases (**C**). Morphology of the liver metastases was altered by 27 Novermber due to changes in contrast agent dynamics (arrow), which is a consequence of the therapy without meaningful changes in size or number (**D**). At diagnosis (7 July 2020), in the retroperitoneal space, lymph nodes (arrow) increased in number and size were detected, indicating lymph node metastases (**E**). At the lower part of the right kidney, the short diameter of the largest node was 20 mm. On 27 Novermber, lymph nodes (arrow) were in regression, the diameter of the largest node decreased to 17 mm (**F**). (**G**,**H**) Compared to the 7 July 2020 baseline (**G**), metastases in the lymph node and the liver (arrow) regressed and morphologically altered, respectively (**H**).

In February of 2020, a 50-year-old white female presented with epigastric pain, sensitivity beneath the right ribs, significant weight loss (8–9 kg), anorexia, diarrhea, and nighttime perspiration. Liver function tests in her blood sample showed increased values (AST: 152 IU/L, ALT: 65 IU/L, GGT: 168 IU/L). Due to the emerging pandemic situation, abdominal ultrasonography was delayed, and eventually, it was carried out in July 2020. Besides the existing ascites, it indicated multiple liver metastases and pathological lymph nodes. An urgently performed abdominal contrast-enhanced computed tomography (CT) on 7 July 2020 indicated multiple liver metastases and suspected primary tumor in the pancreas tail (Figure 1). By this time, her physical condition has severely deteriorated (ECOG: 2). A week later, a core biopsy was taken from one of the liver metastatic sites, and histology revealed a well-differentiated neuroendocrine tumor. Meanwhile, gastroscopy and colonoscopy were also performed and found no malignancy. On 29 July, somatostatin analogue treatment was started (lanreotide 120 mg injection) with continued monthly treatment during the disease. Liver test parameters were: AST:160, ALT:51, GGT:297. The second lanreotide injection was applied on 26 August when liver test values increased despite the therapy (AST: 840, ALT: 467).

In parallel, molecular diagnostics was initiated to identify targeted therapy options. However, after pathological validation, both FFPE tissue samples failed internal quality control due to insufficient tumor content and were excluded from further DNA extraction. Similarly, a previous attempt of the Ki-67 immunohistochemistry assay also failed due to the sample quality. As re-biopsy did not appear feasible, a blood liquid biopsy was taken for cfDNA analysis. 10 mL whole blood was collected in Cell-Free DNA BCT^®^ (Streck) and stored at room temperature. cfDNA was isolated within 3 days using QIAamp MinElute ccfDNA Midi kit (Qiagen). The Oncompass All-In-One comprehensive genomic profiling test was performed, which fully covers the exons of 591 protein-encoding genes and includes 22 gene fusions and 4 promoter regions of key cancer-specific genes, and 24 miRNA genes. The validated cancer panel is optimized both for tissue and liquid biopsy. 

Agilent SureSelect enrichment technology (Human All Exon enrichment) was used for library preparation. Paired-end sequencing was performed on NovaSeq 6000 S2 PE150 XP platform with 2 × 150 bp 15 million read pairs (+/−3%). 

Filtering was carried out by using the QCI (QIAGEN Clinical Insight Interpret 8.0.20210827), according to the following parameters: call quality of at least 30.0 and pass upstream pipeline filtering and read depth of at least 10.0 and allele fraction of at least 1.0 with genotype quality of at least 30.0. Then variants that were observed with an allele frequency of 10.0%≤ of the genomes in the 1000 genomes project or at least 10.0% of the ExAC Frequency or present in dbSNP or of 10.0%≤ of the gnomAD frequency, unless established as pathogenic common variant, were excluded. Benign and likely benign variants (according to computed ACMG guidelines classification) were also excluded. Next, only exonic variants (up to 2 bases into the intron) were retained. In total, 5841 genetic variants were identified, following bioinformatical and functional filtering, 38 exonic variants were retained (Table 1), including *PIK3CA* p.P539R, *TP53* p.C135F, *TSC2* p.E532*, *TSC2* p.P542R, *DAXX* p.E454*, *SMO* p.R726Q, *KDM6A* p.T584M, *PTPRD* p.V892A, and *TET2* p.L34F. Tumor mutational burden (TMB) was low (2 mutations/megabase).

Interpretation of the identified alterations revealed that *PIK3CA* p.P539R is an activating driver mutation according to preclinical evidence [25,26,27]. It is classified as “likely pathogenic” in the ClinVar database, and it is also listed in the COSMIC database of somatic mutations in cancer. *TP53* p.C135F is a loss-of-function variant [28,29,30,31]. In ClinVar, it is classified as “likely pathogenic”, according to the IARC-TP53 database, it is “non-functional” and listed in the COSMIC database. *TSC2* p.E532* and *TSC2* p.P542R are undescribed mutations. E532* is a truncating, nonsense mutation resulting in nonsense-mediated decay (NMD) targeted premature termination codon (PTC) [32]. Thus, the loss of function is highly likely. According to in silico predictions, P542R is classified as either probably damaging [33] or tolerated [34]. *DAXX* p.E454* is another truncating mutation. Although somatic *DAXX* mutations are common in pancreatic neuroendocrine tumors, their therapeutic relevance is obscure. *SMO* p.R726Q has been suggested as a non-driver mutation in gastric cancer [35]. *KDM6A* p.T584M is classified as benign/likely benign, and *PTPRD* p.V892A as benign, respectively, in ClinVar. *TET2* p.L34F is a benign polymorphism [36,37,38]. It is noteworthy that the allele frequency of most variants was high (Table 1), indicating a high ctDNA ratio within the cfDNA fraction. Indeed, the concentration of isolated cfDNA was 8.065 ng/µL, whereas the mean value is 3.33 in our sample set. Moreover, fragment analysis revealed that the cfDNA fraction almost exclusively consisted of fragments of ~160 bp and ~320 bp, approximately the length of DNA wrapped around a nucleosome plus its linker and its dimer, respectively, which is the characteristic size of tumor origin [39]. The ratio of tumor-derived cfDNA can range from below 0.01% to as high as 93% [40], and this wide range in quantity is directly correlated with tumor burden [41,42]. 

All exonic variants were analyzed by our digital drug assignment (DDA) system (RealTime Oncology Treatment Calculator™ v1.64) [24]. Based on the scientific evidence in relation to the molecular profile, the system ranked the phosphatidylinositol-3-kinase (PI3K) inhibitors, alpelisib and copanlisib, and the mTOR inhibitor everolimus as of the highest relevance (Table 2). No resistance mutations to mTOR inhibition (e.g., *KRAS*, *BRAF*, *MYC*, *CCNE1*) were identified. Other pNET-associated targeted drugs, such as the approved multi-tyrosine kinase inhibitor sunitinib, were ranked lower. Based on these results, molecular tumor board discussion in August concluded that everolimus, which is approved in the tumor type and related positively to two important driver mutations of the patient (*PIK3CA* p.P538R and *TSC2* p.E532*), appears to be the most suitable targeted therapy in this case. 

Everolimus treatment commenced on 31 August with 1 × 2.5 mg Afinitor, considering the increased liver test values (AST: 601, ALT: 271, GGT: 335, SeBi: 29 μmol/L). On the same day, abdominal ultrasonography confirmed multiple liver metastases and advanced regional and retroperitoneal lymph node involvement. The liver test values on 7 September were AST: 451, ALT:195, GGT: 387, SeBi: 63. Due to the improving liver test results, the everolimus dosage was increased to 5 mg daily on 11 September (test of the day: AST: 206, ALT: 97, GGT: 322, SeBi: 42), then to 10 mg daily on 17 September. The liver test returned to normal range on 29 September 2020. Her physical condition rapidly improved in response to the treatment. By November, all physical symptoms improved significantly or disappeared (ECOG: 0–1). 

Tumor response was evaluated by CT imaging, which detected regression in lymph nodes and stable tumor in the pancreas (Figure 1). In the pancreas, a 22 × 27 mm, inhomogeneous, oval soft-tissue mass could be observed behind the tail at diagnosis (7 July 2020). This mass could not be distinguished from the outline of the pancreas with certainty. On 27 November, the mass was detected unchanged and of the same size, indicating stable disease. The liver was significantly enlarged at diagnosis, and major parts of the liver area were filled with occasionally conflating hypodense regions of blurred outlines, indicating multiple metastases. By 27 November, morphology of the liver metastases was altered, due to changes in contrast agent dynamics, which is considered a consequence of the therapy without meaningful changes in size or number. At diagnosis, enlarged lymph nodes in increased numbers were detected in the retroperitoneal space, indicating lymph node metastases. Short diameter of the largest node was 20 mm. On 27 November lymph nodes were in regression, diameter of the largest node decreased to 17 mm. Taken together, radiological and liver tests, as well as rapid improvement of physical conditions, all indicated disease control in response to molecularly targeted therapy. 

Nevertheless, on 9 March 2021 increased liver test values were detected again (AST:80, ALT:41, GGT:172, SeBi 5.4). Abdominal ultrasonography on 26 March indicated unaltered liver status, liver tests of the day were further increased (AST: 185, ALT: 107, GGT: 306, SeBi: 73). Due to her weakened condition, elevated liver test results and clinical jaundice, she was hospitalized, but despite all efforts, her general condition failed to improve. Restaging CT on 29 March detected progression both in the liver and lymph nodes, and novel malignancies between the inferior vena cava and the hepatic portal vein and in the peritoneum, prompting palliative care. Her liver tests deteriorated further (5 April: AST:3379, ALT:854, GGT:184, SeBi:184), and with her general condition continuously declining on 4 May she succumbed to the disease.

## 3. Discussion

Although targeted therapies clearly play an increasing role in treating pancreatic neuroendocrine cancer, the choice of the appropriate treatment can be challenging. A decision has to be made among different approved therapies. Yet, an insufficient tissue sample is a common issue, resulting in the lack of molecular information, while on the other hand, complex molecular profiles may present with a plethora of alterations. The ongoing COVID-19 pandemic further complicates oncological care through various effects, including delayed cancer diagnoses due to health care overload or reluctance to attend visits. Here we present a case where personalized, targeted therapy was applied for a belatedly diagnosed, heavily metastatic pancreatic neuroendocrine carcinoma, based on NGS of cfDNA, resulting in immediate response. Finding the right therapy among multiple available approved therapies was crucial, as the rapidly worsening status presented an imminent threat of losing the patient.

Treatment options for pancreatic neuroendocrine tumors have been reviewed by Akirov et al. [43]. For metastatic disease with high disease burden, there are three different main groups of medical therapies available to control tumor growth: somatostatin analogues (octreotide and lanreotide), molecularly targeted treatments (everolimus, sunitinib, and others), and chemotherapies (capecitabine, 5-fluorouracil, dacarbazine, oxaliplatin, streptozotocin, and temozolomide). Effective deployment of molecularly targeted therapies requires individual tumor molecular profile information, which can be limited in pancreatic cancer due to insufficient biopsies. As an alternative, liquid biopsies can provide access to tumor DNA in the form of cfDNA. As the amount of cfDNA increases with increased metastatic burden, this approach appears to be better suited for the metastatic setting. Accordingly, it was possible to isolate cfDNA of tumor origin in high quantity and quality in this case, highlighting the utility of cfDNA analysis for metastatic pancreatic tumors. 

The unfortunate situation due to the COVID-19 pandemic provided unusual circumstances in clinical oncology. Yet, in the present case, the pandemic-inflicted necessity brought about clinical evidence for the utility of large-panel sequencing of cfDNA in advanced pNET. This case thus may indicate a valuable approach in suboptimal situations, where the need for urgent molecular diagnosis and scarcity of tissue samples coalesce.

Recent efforts to utilize liquid biopsy-based technologies in pancreatic cancer have mainly focused on ductal adenocarcinoma [17,18,19], but in neuroendocrine tumors, cfDNA has not been incorporated into routine clinical practice [20]. There are two basic methodologic approaches to cfDNA analysis. Polymerase chain reaction (PCR) based methods, of which droplet digital PCR (ddPCR) has gained popularity for diagnostic and monitoring purposes and next generation sequencing (NGS). ddPCR is more sensitive than most NGS-based methods. However, it is restricted to a few hotspots. In most studies evaluating cfDNA in PDAC, *KRAS* hotspot mutations were tested [17,18,19]. Although NGS provides more comprehensive mutational profiles, using large-panel sequencing in clinical practice is still uncommon. Here we provide clinical evidence that the high-quality cfDNA that is associated with advanced tumors is suitable for large-panel sequencing in advanced pNET. Thus, cfDNA-based comprehensive genomic analysis is a viable approach for personalized treatment stratification of this tumor type. It is noteworthy that two *TSC2* mutations were detected. This gene is not included in many small gene panels, yet *TSC2* loss-of-function profoundly impacted decision-making. 

Genomic assays that enable the identification of genetic alterations in individual tumor samples are increasingly being used in clinical diagnostics as a means of identifying therapeutic options [44]. Moreover, non-personalized targeted therapies were associated with significantly poorer outcomes than cytotoxic agents, which in turn were worse than personalized, targeted therapy [45], highlighting the importance of molecular target matching in indications with more approved targeted therapies. NGS of a 591-gene panel identified several alterations characteristic to pNETs, such as mTOR pathway activating mutations, *DAXX* loss-of-function mutation, and *ATRX* copy loss, as well as several other mutations in various genes associated with tumorigenesis. Some identified mutations are certainly drivers, but not readily targetable (*TP53* p.C135F, *MUTYH* p.Y179C, *DAXX* p.E454*) or non-driver alterations in known driver genes (*SMO* p.R726Q, *KDM6A* p.T584M, *PTPRD* p.V892A, *TET2* p.L34F), and several VUS alterations were also detected. Analysis of the molecular profile prompted mTOR inhibition as the primary targeted approach because of the presence of a well-known *PIK3CA* activating mutation, an obvious *TSC2* loss-of-function mutation, and an additional uncharacterized *TSC2* point mutation, and also because everolimus is approved in this tumor type. This decision was supported by a digital drug assignment (DDA) system, which sorted and ranked all alterations and the associated drugs [24] (Table 1 and Table 2). With the ever-increasing complexities of genomic assays, incorporation of interpretation resources into clinical decision-making is crucial [44], and computational approaches will probably gain more relevance in the future. It is noteworthy that the PI3K pathway is central to somatostatin antiproliferative signaling and AKT1 overexpression was shown to block the antiproliferative action of octreotide in pituitary tumor cells [46], raising a possible explanation for the poor efficacy of somatostatin analogue treatment.

The present case clearly demonstrates the utility of large-panel NGS analysis of cfDNA in advanced pNET for personalized treatment. Such detailed molecular profiling enabled making an informed decision among alternative approved targeted therapies. Interpretation and ranking of the various identified alterations and the associated drugs were aided by the DDA system. Selecting the first treatment is of special importance in the case of advanced disease. This case suggests the importance of prospective studies further exploring detailed molecular profiling of cfDNA in advanced pancreatic tumors and points towards the potential of computer-aided drug assignment.

## Figures and Tables

**Table 1:** Exonic alterations identified by 591-gene panel sequencing of cfDNA.

Position	Gene Symbol	Protein Alteration	CDS Alteration	dbSNP ID	Driver AEL Score *	Allele Frequency (%)
chr3:179218286	*PIK3CA*	p.P539R	c.1616C>G	121913285	70.81	65.00
chr17:7675208	*TP53*	p.C135F	c.404G>T	587781991	53.7	44.03
chr1:45332803	*MUTYH*	p.Y179C	c.536A>G	34612342	12.35	89.92
chr16:2064422	*TSC2*	p.E532*	c.1594G>T		2.68	78.11
chr16:2065544	*TSC2*	p.P542R	c.1625C>G	764191178	2.68	91.11
chr3:37047638	*MLH1*	p.K617N	c.1851G>T		0.61	76.68
chr4:162111346	*FSTL5*	p.E17D	c.51G>C	140747357	0.39	46.97
chr6:33320116	*DAXX*	p.E454*	c.1360G>T		0.34	80.74
chr7:99011389	*TRRAP*	p.A3717T	c.11149G>A	199541716	0.25	45.65
chr20:53576056	*ZNF217*	p.R903Q	c.2708G>A	61748378	0.24	47.70
chr2:140475231	*LRP1B*	p.A3178T	c.9532G>A	72899872	0.21	89.73
chr5:256368	*SDHA*	p.L649fs*4	c.1945_1946delTT	112307877	0.18	13.11
chr12:53215425	*RARG*	p.R115C	c.343C>T		0.05	2.23
chr16:56834405	*NUP93*	p.L567P	c.1700T>C	774760575	0.04	10.78
chrX:67711447	*AR*	p.G644V	c.1931G>T		0.04	78.93
chr3:138946005	*FOXL2*	p.G240S	c.718G>A	767088367	0.01	11.41
chr1:205661956	*SLC45A3*	p.V377M	c.1129G>A	150525587	0	88.35
chr10:17071551	*CUBN*	p.N834D	c.2500A>G	759954219	0	90.93
chr10:86892221	*BMPR1A*	p.Q109K	c.325C>A		0	78.77
chr14:95455532	*SYNE3*	p.R328G	c.982C>G	145141808	0	50.75
chr18:33211925	*CCDC178*	p.V737L	c.2209G>C	117587736	0	52.54
chr19:40843915	*CYP2A6*	p.V456I	c.1366G>A	201305272	0	27.02
chr19:8946760	*MUC16*	p.P10004S	c.30010C>T	200869910	0	40.88
chr19:8958984	*MUC16*	p.S5929F	c.17786C>T	74872724	0	44.66
chr3:187729913	*BCL6*	p.E164D	c.492G>T	61752081	0	91.27
chr3:49897319	*MST1R*	p.R715Q	c.2144G>A	777611015	0	87.90
chr4:59457	*ZNF595*	p.I11V	c.31A>G	6834707	0	16.78
chr5:14291211	*TRIO*	p.E346*	c.1036G>T		0	45.38
chr9:136475373	*SEC16A*	p.Y748S	c.2243A>C	201466249	0	48.75
chrX:1193297	*CRLF2*	p.K258R	c.773A>G	1348007359	0	15.38
chr12:31097896	*DDX11*	p.Q592E	c.1774C>G	2911826	−0.01	1.99
chr7:129212264	*SMO*	p.R726Q	c.2177G>A	142495470	−2.5	47.40
chr7:108180375	*NRCAM*	p.E900G	c.2699A>G	34721383	−5	51.18
chrX:45063645	*KDM6A*	p.T584M	c.1751C>T	141353229	−9.33	89.78
chr9:8486142	*PTPRD*	p.V892A	c.2675T>C	151005956	−9.93	9.66
chr17:65538235	*AXIN2*	p.S390G	c.1168A>G	139871607	−10	48.85
chrX:1196817	*CRLF2*	p.V244M	c.730G>A	151218732	−24.22	100.00
chr4:105234042	*TET2*	p.L34F	c.100C>T	111948941	-53.32	49.29

* AEL: Aggregated Evidence Level, a computed driver evidence score according to the digital drug assignment system [24].

**Table 2:** Prioritized compound list generated by digital drug assignment system, based on the tumor molecular profile.

Compound	Associated Driver(s)	Compound AEL Score *
ALPELISIB	*PIK3CA* p.P539R	406.80
COPANLISIB	*PIK3CA* p.P539R	397.10
EVEROLIMUS	*PIK3CA* p.P539R *TSC2* p.P542R *TSC2* p.E532*	249.20
SIROLIMUS	*PIK3CA* p.P539R *TSC2* p.P542R *TSC2* p.E532*	200.68
BEVACIZUMAB	*TP53* p.C135F	178.03
TEMSIROLIMUS	*PIK3CA* p.P539R	171.96
METFORMIN	*PIK3CA* p.P539R	171.15
ASPIRIN	*PIK3CA* p.P539R	150.57
SUNITINIB		114.69
PAZOPANIB		113.91

* AEL: Aggregated Evidence Level, a computed compound evidence score according to the digital drug assignment system [24].

## References

[1] Young K., Iyer R., Morganstein D., Chau I., Cunningham D., Starling N. (2015). Pancreatic neuroendocrine tumors: A review. Futur. Oncol..

[2] Raymond E., Dahan L., Raoul J.-L., Bang Y.-J., Borbath I., Lombard-Bohas C., Valle J., Metrakos P., Smith D., Vinik A. (2011). Sunitinib Malate for the Treatment of Pancreatic Neuroendocrine Tumors. N. Engl. J. Med..

[3] Caplin M.E., Pavel M., Ćwikła J.B., Phan A.T., Raderer M., Sedláčková E., Cadiot G., Wolin E.M., Capdevila J., Wall L. (2014). Lanreotide in Metastatic Enteropancreatic Neuroendocrine Tumors. N. Engl. J. Med..

[4] Strosberg J., El-Haddad G., Wolin E., Hendifar A., Yao J., Chasen B., Mittra E., Kunz P.L., Kulke M.H., Jacene H. (2017). Phase 3 Trial of ^177^Lu-Dotatate for Midgut Neuroendocrine Tumors. N. Engl. J. Med..

[5] Yao J.C., Shah M.H., Ito T., Bohas C.L., Wolin E.M., Van Cutsem E., Hobday T.J., Okusaka T., Capdevila J., de Vries E. (2011). Everolimus for Advanced Pancreatic Neuroendocrine Tumors. N. Engl. J. Med..

[6] Iyer R.V., Konda B., Fountzilas C., Mukherjee S., Owen D., Attwood K., Wang C., Ma C.W., Minderman H., Ba S.S. (2020). Multicenter phase 2 trial of nintedanib in advanced nonpancreatic neuroendocrine tumors. Cancer.

[7] Capdevila J., Fazio N., Lopez C.L., Teule A., Valle J.W., Tafuto S., Custodio A.B., Reed N., Raderer M., Grande E. (2019). Final results of the TALENT trial (GETNE1509): A prospective multicohort phase II study of lenvatinib in patients (pts) with G1/G2 advanced pancreatic (panNETs) and gastrointestinal (giNETs) neuroendocrine tumors (NETs). J. Clin. Oncol..

[8] Xu J., Shen L., Bai C., Wang W., Li J., Yu X., Li Z., Li E., Yuan X., Chi Y. (2020). Surufatinib in advanced pancreatic neuroendocrine tumours (SANET-p): A randomised, double-blind, placebo-controlled, phase 3 study. Lancet Oncol..

[9] Tirosh A., Kebebew E. (2020). Genetic and epigenetic alterations in pancreatic neuroendocrine tumors. J. Gastrointest. Oncol..

[10] Koschmann C., Lowenstein P.R., Castro M.G. (2016). ATRX mutations and glioblastoma: Impaired DNA damage repair, alternative lengthening of telomeres, and genetic instability. Mol. Cell. Oncol..

[11] Hughes C.M., Rozenblatt-Rosen O., Milne T., Copeland T.D., Levine S., Lee J.C., Hayes D.N., Shanmugam K.S., Bhattacharjee A., Biondi C.A. (2004). Menin Associates with a Trithorax Family Histone Methyltransferase Complex and with the Hoxc8 Locus. Mol. Cell.

[12] Kaelin W.G. (2002). Molecular basis of the VHL hereditary cancer syndrome. Nat. Rev. Cancer.

[13] Thorpe L., Yuzugullu H., Zhao J.J. (2015). PI3K in cancer: Divergent roles of isoforms, modes of activation and therapeutic targeting. Nat. Rev. Cancer.

[14] Janku F., Yap T.A., Meric-Bernstam F. (2018). Targeting the PI3K pathway in cancer: Are we making headway?. Nat. Rev. Clin. Oncol..

[15] Luke J.J., Oxnard G.R., Paweletz C.P., Camidge D.R., Heymach J.V., Solit D.B., Johnson B.E. (2014). Realizing the Potential of Plasma Genotyping in an Age of Genotype-Directed Therapies. J. Natl. Cancer Inst..

[16] Rodríguez J., Avila J., Rolfo C., Ruíz-Patiño A., Russo A., Ricaurte L., Ordóñez-Reyes C., Arrieta O., Zatarain-Barrón Z.L., Recondo G. (2021). When Tissue is an Issue the Liquid Biopsy is Nonissue: A Review. Oncol. Ther..

[17] Kamyabi N., Bernard V., Maitra A. (2019). Liquid biopsies in pancreatic cancer. Expert Rev. Anticancer Ther..

[18] Lee J., Park S.S., Lee Y.K., Norton J.A., Jeffrey S.S. (2019). Liquid biopsy in pancreatic ductal adenocarcinoma: Current status of circulating tumor cells and circulating tumorDNA. Mol. Oncol..

[19] Gall T.M., Belete S., Khanderia E., Frampton A.E., Jiao L.R. (2019). Circulating Tumor Cells and Cell-Free DNA in Pancreatic Ductal Adenocarcinoma. Am. J. Pathol..

[20] Rizzo F.M., Meyer T. (2018). Liquid Biopsies for Neuroendocrine Tumors: Circulating Tumor Cells, DNA, and MicroRNAs. Endocrinol. Metab. Clin. N. Am..

[21] Boons G., Vandamme T., Peeters M., Beyens M., Driessen A., Janssens K., Zwaenepoel K., Roeyen G., Van Camp G., De Beeck K.O. (2018). Cell-Free DNA From Metastatic Pancreatic Neuroendocrine Tumor Patients Contains Tumor-Specific Mutations and Copy Number Variations. Front. Oncol..

[22] Zakka K., Nagy R., Drusbosky L., Akce M., Wu C., Alese O.B., El-Rayes B.F., Kasi P.M., Mody K., Starr J. (2020). Blood-based next-generation sequencing analysis of neuroendocrine neoplasms. Oncotarget.

[23] Raymond E., Thieblemont C., Alran S., Faivre S. (2020). Impact of the COVID-19 Outbreak on the Management of Patients with Cancer. Target. Oncol..

[24] Petak I., Kamal M., Dirner A., Bieche I., Doczi R., Mariani O., Filotas P., Salomon A., Vodicska B., Servois V. (2021). A computational method for prioritizing targeted therapies in precision oncology: Performance analysis in the SHIVA01 trial. NPJ Precis. Oncol..

[25] Dogruluk T., Tsang Y.H., Espitia M., Chen F., Chen T., Chong Z., Appadurai V., Dogruluk A., Eterovic A.K., Bonnen P.E. (2015). Identification of Variant-Specific Functions of PIK3CA by Rapid Phenotyping of Rare Mutations. Cancer Res..

[26] Gymnopoulos M., Elsliger M.-A., Vogt P.K. (2007). Rare cancer-specific mutations in PIK3CA show gain of function. Proc. Natl. Acad. Sci. USA.

[27] Ng P.K.-S., Li J., Jeong K.J., Shao S., Chen H., Tsang Y.H., Sengupta S., Wang Z., Bhavana V.H., Tran R. (2018). Systematic Functional Annotation of Somatic Mutations in Cancer. Cancer Cell.

[28] Dearth L.R., Qian H., Wang T., Baroni T.E., Zeng J., Chen S.W., Yi S.Y., Brachmann R.K. (2007). Inactive full-length p53 mutants lacking dominant wild-type p53 inhibition highlight loss of heterozygosity as an important aspect of p53 status in human cancers. Carcinogenesis.

[29] Jordan J.J., Inga A., Conway K., Edmiston S., Carey L.A., Wu L., Resnick M.A. (2010). Altered-Function p53 Missense Mutations Identified in Breast Cancers Can Have Subtle Effects on Transactivation. Mol. Cancer Res..

[30] Gonin-Laurent N., Gibaud A., Huygue M., Lefèvre S.H., Le Bras M., Chauveinc L., Sastre-Garau X., Doz F., Lumbroso L., Chevillard S. (2006). Specific TP53 mutation pattern in radiation-induced sarcomas. Carcinog.

[31] Goldschneider D., Horvilleur E., Plassa L.-F., Guillaud-Bataille M., Million K., Wittmer-Dupret E., Danglot G., De Thé H., Bénard J., May E. (2006). Expression of C-terminal deleted p53 isoforms in neuroblastoma. Nucleic Acids Res..

[32] Litchfield K., Reading J.L., Lim E.L., Xu H., Liu P., Al-Bakir M., Wong Y.N.S., Rowan A., Funt S.A., Merghoub T. (2020). Escape from nonsense-mediated decay associates with antitumor immunogenicity. Nat. Commun..

[33] Adzhubei I.A., Schmidt S., Peshkin L., Ramensky V.E., Gerasimova A., Bork P., Kondrashov A.S., Sunyaev S.R. (2010). A method and server for predicting damaging missense mutations. Nat. Methods.

[34] Vaser R., Adusumalli S., Leng S.N., Sikic M., Ng P.C. (2016). SIFT missense predictions for genomes. Nat. Protoc..

[35] Wang X.-D., Inzunza H., Chang H., Qi Z., Hu B., Malone D., Cogswell J. (2013). Mutations in the Hedgehog Pathway Genes SMO and PTCH1 in Human Gastric Tumors. PLoS ONE.

[36] Martínez-Avilés L., Besses C., Álvarez-Larrán A., Torres E., Serrano S., Bellosillo B. (2012). TET2, ASXL1, IDH1, IDH2, and c-CBL genes in JAK2- and MPL-negative myeloproliferative neoplasms. Ann. Hematol..

[37] Abdel-Wahab O., Mullally A., Hedvat C., Garcia-Manero G., Patel J., Wadleigh M., Malinge S., Yao J.J., Kilpivaara O., Bhat R. (2009). Genetic characterization of TET1, TET2, and TET3 alterations in myeloid malignancies. Blood.

[38] Nibourel O., Kosmider O., Cheok M., Boissel N., Renneville A., Philippe N., Dombret H., Dreyfus F., Quesnel B., Geffroy S. (2010). Incidence and prognostic value of TET2 alterations in de novo acute myeloid leukemia achieving complete remission. Blood.

[39] Mouliere F., Rosenfeld N. (2015). Circulating tumor-derived DNA is shorter than somatic DNA in plasma. Proc. Natl. Acad. Sci. USA.

[40] Schwarzenbach H., Hoon D.S.B., Pantel K. (2011). Cell-free nucleic acids as biomarkers in cancer patients. Nat. Rev. Cancer.

[41] Jahr S., Hentze H., Englisch S., Hardt D., Fackelmayer F.O., Hesch R.D., Knippers R. (2001). DNA fragments in the blood plasma of cancer patients: Quantitations and evidence for their origin from apoptotic and necrotic cells. Cancer Res..

[42] Diehl F., Li M., Dressman D., He Y., Shen D., Szabo S., Diaz L.A., Goodman S.N., David K.A., Juhl H. (2005). Detection and quantification of mutations in the plasma of patients with colorectal tumors. Proc. Natl. Acad. Sci. USA.

[43] Akirov A., Larouche V., AlShehri S., Asa S.L., Ezzat S. (2019). Treatment Options for Pancreatic Neuroendocrine Tumors. Cancers.

[44] Berger M.F., Mardis E.R. (2018). The emerging clinical relevance of genomics in cancer medicine. Nat. Rev. Clin. Oncol..

[45] Schwaederle M., Zhao M., Lee J.J., Eggermont A.M., Schilsky R.L., Mendelsohn J., Lazar V., Kurzrock R. (2015). Impact of Precision Medicine in Diverse Cancers: A Meta-Analysis of Phase II Clinical Trials. J. Clin. Oncol..

[46] Theodoropoulou M., Stalla G.K. (2013). Somatostatin receptors: From signaling to clinical practice. Front. Neuroendocr..

